# The Medical Aspect of Boswell's "Life of Johnson," with Some Account of the Medical Men Mentioned in That Book

**Published:** 1910-12

**Authors:** Bertram M. H. Rogers

**Affiliations:** Physician to the Bristol Royal Hospital for Sick Children and Women.


"Scire est nescire, nisi id me
Scire alius sciret."

DECEMBER, 1910.

### THE MEDICAL ASPECT OF BOSWELL'S "LIFE OF JOHNSON," WITH SOME ACCOUNT OF THE MEDICAL MEN MENTIONED IN THAT BOOK.

The Presidential Address, delivered on October 12th, 1910, at the opening of the Thirty-seventh Session of the Bristol Medico-Chirurgical Society.

BY

Bertram M. H. Rogers, M.D., B.Ch., B.A. Oxon.,

*Physician to the Bristol Royal Hospital for Sick Children and Women.*

A year ago, when you the members of this Society elected me to the honourable position of President-Elect, I said that I hoped to have an opportunity of expressing my appreciation of the honour you had done me in more adequate terms than I was then able to muster. The time has come for me to take the chair at our monthly meetings, and my first duty is to say how much I am indebted to you for your kindness in placing me there. We all indulge in ambitions of some sort during our lives, but none can be a more honourable one than to hope that some day one may be thought of sufficient importance in our own profession to be placed at the head of its Society. I am very sensible of the honour that you have done me. If I

have not in other ways reached the heights of my ambition, I have at least attained one which I venture to believe shows that I stand well with my professional brethren.

The next duty that I have to perform is of almost as pleasant a character, to read you a presidential address. From the very circumstances of the case one must plead inexperience. It falls to few of us to occupy a presidential chair twice, but to prepare an address which will be of interest to you (if difficult) is by no means an unpleasant task.

Of recent years it has become the custom, in giving presidential addresses, to depart somewhat from the severely professional subject and to select one which, though of medical interest, will also suit those of literary or antiquarian tastes, and I propose this year to address you on such medical topics as may be found in one of the greatest books in our language, Boswell's *Life of Samuel Johnson.* In doing so I must ask your indulgence if at times I appear to depart somewhat from the strict lines that I have laid down for myself, for I have found in the preparation of my paper during the last twelve months many matters of medical gossip which I thought would be of as much interest to you as they were to me.

I propose to marshal my facts under three heads. To treat first with Johnson's own life as depicted by himself or Boswell from a medical aspect; secondly, to give a sketch of his opinions and experience of medical practice of that time; and thirdly, to refer, only in brief outline, to a few of the numerous medical men with whom Johnson came in contact and enjoyed his friendship.

When reading the vivid account that Boswell gives of his great patron, one cannot help being struck with the great amount of bad health and physical suffering that the "great lexicographer" endured. When in his seventy-fifth year he wrote to his life-long friend Hector, a surgeon at Birmingham, that his health from his twentieth year had been such as seldom afforded him a single day's ease, one is forced to the conclusion that many of the self-accusations that he makes in his *Prayers and Meditations* of dilatoriness and laziness were due to this reason.

Dr. Samuel Johnson was one of two sons, his brother Nathaniel dying when twenty-five years old. Boswell ascribes Samuel's constitutional weakness to the fact that he was the son of a man of fifty-six by a wife of forty years of age, rather than to any hereditary reason, for his father, Michael Johnson, was a man of large and robust frame.

The poor little Samuel was in early life attacked with scrofula, and was one of the last persons in this country to be touched by royal hands for the King's Evil. This remarkable ceremonial, for which a special service existed in the Book of Common Prayer till 1719, had been in vogue since the time of Edward the Confessor, and we marvel in the present day at the persistence of what one monarch of this realm called "a silly superstition." Johnson was touched by Queen Anne, whom he faintly remembered as a "lady in diamonds and a long black hood," and was presented with the token or coin which I understand is still preserved in the British Museum. This ceremony probably took place in Lent, 1712, as an advertisement is to be found in the *London Gazette* of that date to say that the Queen was ready to touch for the Evil. Johnson was then twenty months old, and was touched on the advice of Sir John Floyer, a physician of Lichfield.

What was the state of the cervical glands at the time of the touching is not recorded, but Johnson bore the scars in his neck all his life. Boswell ascribes Johnson's defective sight to the scrofula, and I think on this ground alone we may assume that Boswell was not a pathologist. It is extremely difficult to say from what defect of the eyes Johnson suffered. In one the sight was very deficient, or as he jocosely remarked, "This dog was never good for much," but one or both appear to have improved as he got older, suggesting that he had myopia, which improved as he became presbyopic, for he says in 1756 that his sight was restored to him. As an instance of how defective it was I may mention one incident. He had promised Mrs. Abingdon to be present at her benefit at Drury Lane Theatre, and he accordingly went and sat in "the front boxes with a body of wits." From this position he could neither hear nor see anything of the play, but he with due courtesy to the great actress sat the piece out—a play of five acts and a farce of two—"wrapped in the gravest abstraction."

For the eye trouble the treatment then employed was of a more active nature than for scrofula, for an issue was cut in his neck, that is an artificial ulcer was made and kept open by the insertion of some irritating substance. To go no farther back than Mr. Augustin Prichard, it is interesting to find an issue claimed as a successful remedy. He states in his reminiscences that his father, the distinguished ethnologist, originated the plan of making a long incision in the scalp for brain diseases, commenting that though a strong remedy, it was sometimes undoubtedly the means of saving life. Even in my student days it was mentioned in the lectures on Materia Medica.

Of his infant and adolescent ailments—common to all flesh—no mention is made. We have only a description of his appearance, which both as a young man and an old one was, to use a moderate expression, peculiar. As a young man at the age of twenty-five, we have an account from the hand of Miss Lucy Porter—the daughter of Johnson's wife by a previous husband—describing him when paying his addresses to his future wife, then double his age, "He was lean and lank, so that the immense structure of his bones was hideously striking to the eye, and the scars of his scrofula were deeply visible, . . . and he often had, seemingly, convulsive starts and odd gesticulations which tended to one's surprise and ridicule." We must be glad that Mrs. Porter overlooked these physical defects in the qualities of the man, for she said, "This is the most sensible man I ever saw in my life."

Boswell, as usual, had his theories about these convulsive starts, and compares them to St. Vitus's dance, and this statement has lead an American physician to assume that Johnson really had this complaint, and traces his subsequent heart troubles to this disease. Johnson had a much simpler explanation, for when asked by the *enfant terrible* of the day why he made such gestures, he replied, "From bad habit, my dear; do you take care to avoid bad habits."

Of his appearance in later life we may draw a general idea from the numerous portraits that are in existence, though no doubt the artist would gracefully and tactfully omit to make a striking feature of anything that would offend. Unfortunately, Johnson was not particular in his personal appearance, or indeed in the niceties of polite behaviour. There is no need to recall them, they do not come within the scope of my address, but one cannot help reminding you of a witty saying of Mrs. Boswell's, who, when no doubt a little out of temper with Johnson's uncouth manners, chided her husband with his servility to the great men, "I have heard of a man leading a bear, but never of a bear leading a man." Johnson's enemies, and for the matter of that his friends, did not mind making coarse remarks about his peculiarities. "The Caliban of Literature," and "The Puffy Pensioner," are not polite, although clever and to the point.

Johnson's chief trouble during his life was a tendency to melancholia and depression of spirits. He claimed that it was hereditary, for his father suffered from it. There are frequent references all through the life of Johnson to this "constitutional melancholia," which he himself termed vile, and which he says made him "mad in life, at least not sober." It caused him when the fit was upon him to shut himself up and avoid the society of his friends. It is related how on one occasion he was found "in a deplorable state, sighing, groaning, and talking to himself, and restlessly walking from room to room." He does not appear to have been averse to talking about it with Boswell, whom he found a sympathetic listener and fellow-sufferer, though the difference between the two men is illustrated by the fact that Johnson's plaints were written in his *Prayers and Meditations,* intended for his own reflection, while Boswell makes his confessions in letters to Johnson, and publishes them unblushingly in the *Life of Johnson.*

Johnson's anxiety about these fits of depression was that he might be deprived of his reason, a not unnatural fear to a man of great mental capacity, whose livelihood, as well as the care of several distressed females and an eccentric doctor, depended on his retention of his faculties. Yet with this constant depression of spirits and general feeling of ill-health, he had the greatest contempt for anyone who paraded his feelings, wrote about them in correspondence, or introduced the subject of his health as an excuse for omitting to write and supply some information asked of him. He could console, but on one occasion his patience was too severely tried by Boswell, and he gave him what may be called a piece of his mind in an unmistakable manner. "You are always complaining of melancholia, and I conclude from these complaints that you are fond of it. No man talks of that which he desires to conceal, and every man desires to conceal that of which he is ashamed. Do not attempt to deny it, *manifestum habemus furem*, make it an invariable and obligatory law to yourself never to mention your own diseases—if you are never to speak of them, you will think on them but little, and if you think on them but little they will molest you rarely. When you talk of them, it is plain that you either want pity or praise ; for praise there is no room—and pity will do you no good. Therefore, from this hour speak no more, think no more, about them." It is significant that Boswell, though candid enough to insert this letter (a gem in its way), makes no comment upon it.

Boswell did not repeat all Johnson's complaints, for Mrs. Thrale says that he was constantly doing so to her ; this was after the great quarrel between them, and Mrs. Thrale may have said it in spite.

I am not aware that we at the present time claim melancholia as a peculiarity of this island, but Dr. Cheyne, whom Boswell describes as "learned, philosophic and pious," wrote a book on this condition, and called it *The English Malady*. Johnson recommended Boswell to study the book, as he would find some useful advice in it.

With all his bad health, Johnson had the greatest contempt for what he calls valetudinarianism, a word that I cannot help thinking has changed its meaning since Johnson's day. According to the dictionaries of the present day it is defined as a state of ill-health, but this will not accord with Johnson's own example, which would be better explained by the modern word neurasthenia. To his friend Hector he says, "Don't grow like Congreve, nor let me grow like him when you are near me," for this same Congreve, an old school-fellow of Johnson's, had grown afraid "to go into any house but his own," and was encouraged to drink by an elderly female he called his cousin, "in which he is very willing to be encouraged," making him always "muddy." And in another place he says, "There is nothing against which an old man should be so much on his guard as putting himself out to nurse. I do not know a more disagreeable character than a valetudinarian, who thinks he may do anything that is for his ease, and indulges himself in the grossest freedom. Sir, he brings himself to the state of a hog in a stye."

Boswell tells us that when he left Johnson in May, 1783, it was with a "fearful apprehension of what might happen before his return, as his health was in a more precarious state than at any time when I had parted with him." I do not think that Boswell made any claim to the reputed powers of second sight that the inhabitants north of the Tweed are often credited to possess, but his fearful apprehensions were fulfilled in a remarkable way. Eighteen days later Johnson had a stroke, and it is of so interesting a character that I will give you rather a full account of the course of the disease. From Johnson's own account we learn that he went to bed as usual after a not very fatiguing day, having only sat for his portrait, which was being painted by Miss Reynolds. After a short sleep he awoke and sat up, when he felt a confusion and indistinctness in his head, which lasted, he supposed, about half a minute. "I was alarmed, and prayed God that however He might afflict my body He would spare my understanding. That prayer, that I might try the integrity of my faculties, I made in Latin verse. The lines were not good, but I knew them not to be very good, but I made them easily, and concluded myself to be unimpaired in faculties." How many of us could compose a prayer in Latin verse under such circumstances—or any other?

"Soon after I perceived that I had suffered a paralytic stroke, and that my speech was taken from me. I had no pain and so little dejection in this dreadful state that I wondered at my own apathy, and considered that perhaps death itself, when it should come, would excite less horror than now seemed to attend it. In order to rouse the vocal organs I took two drams of wine. Wine has been celebrated for the production of eloquence. I put myself in violent motion and I think repeated it [probably the wine], but all was in vain. I went to bed and, strange as it may seem, I think slept." On awakening he found that his power of writing was not lost, so wrote instructions to his servant Francis and a friend, though he found some difficulty. "My hand, I know not how or why, made wrong letters." How rapid was his recovery will be seen when I tell you that the account I have just read to you was written by him only two days after the stroke, and further he says that he was able to repeat the Lord's Prayer, "with no very imperfect articulation."

The clearness of this account and the presence of mind under the circumstances are remarkable, no less remarkable too are Johnson's methods of treatment. Two drams of wine to produce eloquence when the speech centre is affected amuses us in the twentieth century, but it will illustrate better than any words of mine the state of darkness in which medical science was in the Georgian Era. In another letter written on the day of his stroke he suggests that by a "speedy application of stimulants much might be done. I question if a vomit, vigorous and rough, would not rouse the organs of speech to action." As a help to the Doctor whom he called in—Dr. Heberden—he says he has been bled frequently for an asthmatic complaint, but had forborne for some time by Dr. Pepys's persuasion, who had perceived that his legs had begun to swell. We are not told any more details of the illness. Boswell was not in London at the time ; had he been we should probably have had much more information.

Johnson made a complete recovery in a very few days, for on July 3rd—that is only seventeen days from the date of his stroke —he writes that "Nature had renewed its operations," and that he could carry on a conversation, though only for a short time, as his nerves were weak. His physicians pronounced him cured, but I fancy he must have taken matters a good deal in his own hand, for he had been for a drive to Hampstead, no small thing in a Georgian carriage, and had even dined at the club.

He now kept well for a considerable time except for attacks of gout, till he was threatened with a surgical operation. The complaint from which he suffered was a sarcocele, a solid swelling as distinguished from a hydrocele, a fluid one.

The surgeons who were called in to pronounce upon the nature of the disease were Percival Pott and Cruikshank, the latter of whom he had previously recommended as a lecturer to the Royal Academy in place of William Hunter. We are not told much, though Boswell says that he has several letters filled with "unpleasing technical details." Whatever was the nature of the disease matters little, for Johnson was spared the ordeal of an operation by the trouble getting well spontaneously. He showed great fortitude in preparing himself for what must have been in those days a grave risk to life, apart from the pain of an operation in the pre-anæsthetic time.

Though Johnson's health was far from satisfactory, he continued to make visits to his friends, and even projected a trip to Italy, but his letters show that he suffered a great deal from his "asthmatic affection and from gout." In May he suddenly obtained extraordinary relief. He had shut himself up and employed the day in the particular exercise of religion—fasting, humiliation and prayer—and his deeply religious belief made him ascribe the sudden amelioration of his symptoms as a divine answer to his prayers.

As Boswell, who had been in London on one of his visits, was returning to Edinburgh, Johnson asked him to lay the details of his case before the best physicians of that city, who at that time, as now, stood high in repute in the medical world. What he hoped to gain from them and their opinion is difficult to say, for they could only form a diagnosis or suggest treatment on the evidence of a non-medical man. But true to his promise,

Boswell duly laid Johnson's case before Drs. Cullen, Hope, Munroe, Gillespie, and Sir Alexander Dick. What their opinions were is not recorded, but Johnson was profoundly obliged to them, possibly because they wrote such flattering encomiums on his greatness. Like most of us—like the least of us—Johnson was susceptible to flattery, even if a little fulsome. Of remedies suggested only one is preserved for us, that of Sir Alexander Dick, who appears to have recommended some rhubarb, cultivated in his own garden in Prestonfields ; this Boswell was to bring when he returned to London.

In the year 1783, when Boswell was in London, in March, it was evident that Johnson was, to use a common expression, breaking up, though his ardour for literature was not abated, and he worked as hard as ever in his more or less dilatory manner. Boswell notes his pallor, shortness of breath and bad nights, and mentions as an instance of how difficult a patient he was that Sir Lucas Pepys, the Thrales' doctor, said, " If you were tractable, sir, I should prescribe for you." Johnson was then taking opium for producing sleep. I believe that this was then the only hypnotic drug known, as chloral and bromides had not been discovered. No doubt Johnson felt his own intractableness, for he said, " How few of one's friends' houses would a man choose to be at when he is sick." And Boswell says that he only mentioned one, the Thrales'.

The poor old man's last days appear to have been very heavy ones for him, for a second attack of gout came upon him " with a violence I never experienced before." The opinion of Dr. Mudge may have relieved his mind somewhat, for he represented the attack of gout as antagonistic to the palsy.

Added to all the troubles of bad health, he had the misfortune to lose, one after another, the members of his strange household. Levet's death, followed by that of Mrs. Williams, left him very solitary. He says, " Sickness and solitude press me very heavily. I could bear sickness better if I were relieved from solitude." You all recollect Johnson's definition of a certain man—he was clubbable, and to no one could that adjective be better applied than to Johnson himself. He loved the society of his fellow-man, and though through his last illness his friends were constant in their visits to him, he felt the loneliness of his own home and the absence of his house companions.

At the end of the year 1783 he was seized with a spasmodic asthma of such violence that he was confined to the house in great distress, sometimes being obliged to sit up all night. Dropsy began to develop, and reached up to his thighs, but was relieved later by a natural evacuation. In April he writes that his "recovery is such that neither myself nor the physicians at all expect," but for all that he was able to get out to the club, and even later in the year to pay a visit to his beloved Oxford, "the magnificent and venerable seat of learning, orthodoxy and Toryism," as Boswell called my native city, an appreciation that might be passed even now except, perhaps, as regards orthodoxy.

We medical men sometimes, and perhaps with justice, complain that our efforts on behalf of our patients meet with scant recognition, or only with thanks of an ephemeral character. I am glad to say that, trying patient as he must have been, Johnson expressed his gratitude in no measured terms for the assiduous attention of his medical attendants. "If the virtue of medicine could be enforced by the benevolence of the prescriber, how soon should I be well," he wrote to his medical man, Dr. Brocklesby.

And so matters went on, asthma, dropsy, and sleeplessness followed him wherever he went, and on his return to London in November it was evident that he could not live long. So greatly had the dropsy increased that incisions had to be made, incisions that Johnson did not think deep enough, and so deepened for himself, thereby rendering himself open to the charge of attempting to hasten his own end. All was of no avail, and having asked his friend Brocklesby to give him a direct answer whether he could recover, and being told he could not without a miracle, he said, "Then I will take no more physic, not even opiates, for I have prayed that I may render up my soul to God unclouded." A few days later he died, passing away so peacefully that his attendants hardly noticed when dissolution took place. Had he lived in these days no doubt he would have had the administrations of a skilled attendant to ease his last hours. He, poor man, had to put up with one that he said was " an idiot, he is as awkward as a turnspit when first put into a wheel, and as sleepy as a dormouse." I am glad to say the sleepy one was dismissed, and a more wakeful one found, to whom Johnson gave his grateful thanks.

It is by no means an easy matter at the present time to form an opinion as to what was the complaint from which Johnson suffered, and which ultimately caused his death. We read of his asthma, the swelling of his legs and breathlessness, but we must not judge too hastily of the terms that were used in those days. They did not recognise the differences between renal, cardiac or bronchial asthma; and the word gout was used in a very loose manner, much the same as many of our own patients make use of it now; and the swelling of his legs may have been due to heart or renal troubles. We must remember too that Boswell was not a medical man, but an advocate, and though he was a well-read man, probably knew little of medicine; indeed, the remarks that he often makes show that he was not more skilled in it or more versed in the diagnosis of disease than the common herd. We must also recall the fact that diagnosis by auscultation was unknown till many years after Johnson's death, and the few drugs that we read of Johnson taking give us no clue as to the diagnosis of the physicians who were attending him.

On this subject two medical men of the United States have given their views. Dr. Cahill believes that Johnson had mitral regurgitation following several rheumatic attacks, more particularly after the one in 1763. I have read through the record of this year with a view of reconciling this suggestion, but am quite unable to accept his theory. This was the year that Boswell made Johnson's acquaintance, and began to collect the notes with the intention of writing the biography, and I cannot believe that he would at the very beginning of his friendship with his great patron have made no reference to an attack of rheumatic fever. I can find no reference that can in any way be made to read that Johnson had such a disease. Dr. Cahill says that Johnson early developed chorea (though he qualifies it by saying that it was habit chorea, a disease that I never have heard produced cardiac disease), and then on this very slender foundation says that the whole of the subsequent medical history may be followed: "The succession of chorea, rheumatism, cardiac dropsy form a clear picture with which all physicians are unhappily too familiar."

The second writer says that he had angina. I cannot agree with this either. It is true that he was attended by the doctor who first described that disease, Dr. Heberden, but I think there is not sufficient evidence for this assumption, although angina was called asthma Heberdenii.

My opinion is that Johnson suffered from asthma as he grew older, and that led to dilatation of the heart with dropsy, and that he died from heart failure. It is quite possible too that there was a good deal of fatty infiltration of the heart muscle, as we know Johnson became very fat in his later years. [1]

I now come to the second part of my paper, and hope that you will bear with me while I give a short account of some of Johnson's opinions on medical matters, and say a few words about the position of medical men and medical treatment as shown in the *Life of Johnson.*

Johnson, as you probably know, was a man who had little sympathy with the fads, and I regret to say sometimes with the opinions of others, and when he disagreed he spoke his mind in terms that could not be mistaken. He for instance was not a believer in the influence of the weather on the health, and he was wont in his rather forcible manner to ride rough-shod over those who expressed opinions differing from his own. "He had," says Boswell, "a contempt for the notion that the weather affects the

[1] Since reading the above my attention has been called by my friend Dr. H. Rolleston to an account of an autopsy on the body of Johnson. I hope to add a condensed statement of what was found to the end of this address. Meanwhile I will only say that my opinion here stated must be modified.

human frame," though in the last year of his life, when writing to Dr. Burney in August, he says, " The weather you know has not been balmy. I am now reduced to think, and am at last content to talk of the weather. Pride must have a fall." The condition of the weather was to him such a self-evident fact that he said, " You are telling us that of which none but men in a mine or a dungeon can be ignorant. Let us bear with patience and enjoy in quiet such elementary changes, whether for the better or worse, as they are never secrets." On one occasion he and Boswell were leaving the "Mitre," where they had been dining, and found it to be raining, and the latter made some commonplace remark on the relaxing effect of rain on the nerves and the depression of spirits that it caused, but admitted it was good for vegetables. " Why yes, sir, it is good for vegetables, and good for the animals who eat those vegetables, and good for the animals who eat those animals."

I presume that I may regard the taking of or abstention from alcoholic fluids as a medical question, for it is one that was fought almost as strongly in Johnson's day as now. It was a drinking age, I might say almost a drunken one. Johnson tells us that even in his day persons were drinking less than their ancestors did, for he remembered when all the decent people of Lichfield got drunk every night, and were none the worse thought of. The drink then (in the days of his ancestors) was chiefly beer, which was cheap, for when wine became commoner the drinkers were not in such " haste," which according to Johnson was the cause of this change in manners. As a young man Johnson took his wine, and could do his bottle, his favourite one being port sweetened with sugar, and he celebrated his acquaintance with Boswell by performing this feat, though he tells us that if he took too much he went home quietly. As a middle-aged man Johnson became an abstainer. He did so from no dislike of wine or because he thought it was wrong to take it, but simply because he was advised to do so by one of his doctors, but by whom or when the advice was given is not recorded.

Johnson once told Boswell that he when on a visit to University College, Oxford, drank three bottles of port at a sitting, and was none the worse for it. The giving up of wine to him was a diminution of pleasure but not of happiness, as there was more pleasure in being rational than drunk. Boswell was none too sparing in his potations, and the doctor was very firm in his advice to him: "I must entreat you to be scrupulous in the use of strong liquors. One night's drunkenness may defeat the labours of forty days well employed." Boswell reminded him how they used to have headaches after the wine they took, but Johnson replied, "No, sir, it was not the wine that made your head ache, but the sense I put into it." Boswell, "What, will sense make a head ache?" Johnson, "Yes, sir, when it is not used to it."

As an instance of what men could drink in those days I may instance the case of a Dr. Campbell, I believe a divine, who stated that he drank thirteen bottles of port at one sitting. Boswell vouched for Campbell's statement, qualifying it with the remark that the stiting was a long one. When discussing this vinous feat Johnson upheld Campbell as a veracious man with pen and ink, "but you could not entirely depend on anything that he told you in conversation, or if there is a fact mixed with it. However, I love Campbell, he was a solid orthodox man, he had a reverence for religion, and though defective in practice, he was religious in principle." Please mark the fine distinction.

The wine consumed in those days was chiefly port, which cost only 5s. 6d. a gallon. Of claret, though not a newly imported wine, Johnson had a very poor opinion, saying a man must be drowned in it before it made him drunk, so that it seems as if the potency of the wine was the measure of its quality rather than the flavour of its bouquet. Everyone knows the famous Johnsonian saying after being induced to taste a glass of claret, "Claret for boys, port for men, but he who aspires to be a hero must drink brandy." I may mention that Johnson would not let Boswell drink the king's health in whisky, but we must bear in mind his aversion to everything Scotch. Florence wine—I presume much the same as the Chianti of the present day—he thought poor stuff. "It is wine only to the eye; it is wine neither while you are drinking it, nor after you have drunk it, it neither pleases the taste nor exhilarates the spirits."

Johnson, who had learned a good deal of medicine from the assistance that he had given Dr. James in compiling his *Dictionary of Drugs*, had his own ideas on medical subjects. This assumption of knowledge on matters not connected with our own particular branch of human learning is a common failing, but perhaps it is not more universal or more firmly established than when connected with our own profession. It constitutes one of the chief difficulties with which all practitioners of medicine have to cope, and much as the art of medicine may advance, the omniscience of the public will keep pace with and even out-run the knowledge of those who make it their life's study. Numerous examples might be given of Johnson's opinion on medical matters, but I will only repeat one, as it bears out the well-known fact of the contagiousness of the common cold in the head. When planning with Boswell the tour to the Hebrides he praised the "magnanimity" of Macaulay in asserting the wonderful story of how, when a ship called at St. Kilda, all the inhabitants were seized with a cold, because it was a well-attested fact. Johnson's theory was that a north-east wind was essential before a stranger could land, owing to the difficulties of the exposed harbour. "The wind," he said, "not the stranger occasions the epidemic cold." I may say that St. Kilda is not one of the Faroe Islands as an American lately stated, but is one of the outlying islands of the Hebrides, and part of the United Kingdom. Johnson's theory of the evil effects of a particular wind is interesting, and probably the reverse of the truth.

On one occasion when a pretty large circle of friends had met Johnson gave his views on medicated baths, a system, if I may call it so, that had been made popular by a certain Dominicetti. Johnson believed them to be of no more value than warm water, having no more effect than tepid moisture. Johnson was not in one of his polite moods that evening, and on one of the members combating this opinion, he said, "Well, sir, go to Dominicetti, and get yourself fumigated, but be sure that the steam be directed to thy head, for that is the peccant part." Cibber said of Johnson, "When his pistol misses fire, he knocks you down with the butt-end of it." A not unfit comment.

One is surprised to find that Johnson had his views on the management of hospitals. "Though many men are nominally entrusted with the administration of hospitals and public institutions, almost all the good is done by one man, by whom the rest are driven on, owing to confidence in him and indolence in them." I am afraid that the converse is also true.

When getting on in life he no doubt felt that there was a fear that people might say of him that his intellectual powers were failing. "There is a wicked inclination in most people to suppose an old man decays in his intellect. If a young or middle-aged man, when leaving a company, does not recollect where he laid his hat, it is nothing; but if some inattention is discovered in an old man, people will shrug up their shoulders and say his memory is going." This is, I fear, an apology for old age.

The belief that disease is the scourge of the Almighty to try our faith no doubt had its origin from the Old Testament and from the Book of Job, in which you will remember the conference between the Almighty and the Evil One on the power of the latter to make the unfortunate victim of disease and misfortune curse God and die. Johnson, a Tory with the orthodox opinions of the time, held views somewhat of this sort: "Acute diseases are the immediate and inevitable strokes of heaven, but of them the pain is short and the conclusion speedy. Chronical disorders, by which we are suspended in tedious tortures between life and death, are commonly the effect of our own misconduct and intemperance. To die is the fate of man, but to die with lingering anguish is generally his own folly."

Johnson was not unmindful of the need of care for the preservation of health. Cleanliness was not much in vogue in those days, and no one can accuse Johnson of the too lavish use of water externally; but the following schedule is of interest, as showing that he had got hold of some sensible rules for health. They were given to Mr. Perkins, Thrale's manager:—

"(1) Turn all care out of you head as soon as you mount your chaise.
(2) Do not think about frugality, your health is worth more than it can cost.
(3) Do not continue any day's journey to fatigue.
(4) Take now and then a day's rest.
(5) Get a smart sea-sickness if you can.
(6) Cast away all anxiety, and keep your mind at ease."

This last direction is the principal; with an unquiet mind neither "exercise nor diet nor physic can be of much use."

It will, I think, not be devoid of interest it we for a few minutes take the measure of the medical profession in the days when Johnson lived and George III was king, and it must be confessed that, though individual medical men were held in high repute, made large incomes, and had honours conferred upon them as now, the state of medical knowledge was in those days deplorably low. None of the great discoveries had been made that at the present time assist us in diagnosing our patient's ailments. The reason for this is not far to seek. Physical science was unknown, undiscovered. It was not till the commencement of the nineteenth century that the foundations of chemistry were laid, or the correct interpretation of the laws of physical science were understood. Anatomy, it is true, had reached a high degree of perfection, but beyond the discovery by Harvey one hundred years before, the physiology of the body was unknown, the functions of the organs misinterpreted, and the causes of disease an unknown quantity. Therapeutics had hardly emerged from the darkness of the days of hideous and disgusting medicaments, and the few reliable drugs were difficult and expensive to obtain. Added to this, the education of a medical man was of the most meagre description. There was no Medical Act to regulate or control the profession, no supervision of examinations of licensing bodies, while the exclusiveness of the Royal College of Physicians had a most harmful effect on the practice of medicine.

Any reference to this period of history would be incomplete without some few words on the theories of medicine that obtained both in this country and on the continent. The remarks must be very short, for it is impossible to go deeply into them owing to their complexity and obscureness. At the beginning of the century the theories of Boerhaave were almost universally accepted and known as the Eclectic System, though they were founded mostly on still older ones, even on Galen's. Health, disease, and debility depended on "tense, relaxed or weak fibre." He adopted the word "putridity" from the pneumatics and the Hippocratic "enormon" as the cause of "motion," an unknown something, which occasions the senses and "motions." Hoffman introduced the Mechanico-Dynamic System, and was followed by Cullen. In his system the nerves played the most important part, nerve force and activity being the terms, and used without any definition. Fever was due to diminished energy of the brain, and was a reparatory effort of Nature. Gout was due to atony of the stomach, and scrofula to acidity, and putrid fever to the humours. In fact, the extraordinary jumble of words without meaning reads very like Christian Science of the present day.

The School of Montpellier was inaugurated by de Bordeau, the originator of the saying, "The tripod of life." In this system the heart, brain and stomach regulated all the other organs, disease being due to acidity of the glands. This was followed by Reil's doctrine of vital force, each organ having its own liability to changes due to season, age, and even the time of day; and to call forth this vital force certain imponderables were required, such as heat, light, electricity. From this Mesmer developed his theory of animal magnetism, and the later romancers the phlogistic and antiphlogistic theories. Lastly, Brown started the theory known as the Brunonian, in which all vital phenomena were due to irritation. Brown's therapeutics are said to have killed more men than the French and the Napoleonic wars put together.

If we are at the present time troubled with quacks, herbalists, bone-setters, prescribing chemists and irregular practitioners of medicine, the condition of affairs in the Georgian days was far worse. Besides numbers of charlatans of all descriptions, three sets of persons carried on practice, uncontrolled, in opposition to the legitimate practitioners of medicine. Firstly, the apothecary. He was little removed from the quack. He was, it is true, in possession of some sort of licence to practise, but his standard of ethics was low and the knowledge of his craft small. At times he was a great asset to the medical man, for he would call him in consultation on treatment. In this way he would be in a position of giving patronage, and so was a person not to be slighted, but rather to be made much of. The herbalists were quite irregular practitioners, they had no qualifications of any sort, and a perusal of their pharmacopœia shows how colossal was their ignorance and immense their impudence. The quack, " a person making a pretence of medical knowledge," was a flourishing person. He was not ashamed to stoop to any artifice that would bring grist to his mill, and entice the public only one degree more ignorant than he, to entrust their bodily ailments to him. The modern quack, and the disbarred practitioner of to-day, is a poor descendant of the Georgian one, they have neither the impudence that can cloak ignorance nor the powers of self-laudation that carry conviction.

We must not form our conceptions of the position of medical men on the success of any well-known man of those days, on the fortunes of Radcliffe or Mead. The bulk of the profession in those days was far worse off from a monetary point of view than we are at the present ; and if the social status of medical men to-day is superior to what it was one hundred and fifty years ago, it is due to our superior education and greater skill, and to our having been able to convince the public by the application of our knowledge that our opinion is worth having and worthy of being acted upon.

To become a successful doctor in Johnson's day a definite course had to be followed. The aspirant to medical distinction had to decide to which political party he would ally himself, and to assiduously toady to the leaders and wire-pullers. Politics ran even higher in those days than they do in the present, and anyone wishing to advance in medicine had better not adopt the

Whig programme. Johnson himself was a Tory of the deepest hue, and could see no good, nor would hear it, of his political opponents. Having decided on the party you wish to be attached to, you must frequent the coffee-house favoured by that side you have selected, be seen there daily, and "learn and practise every obsequious and crawling art which dishonours a man, and so by slow degrees to attract and secure patrons." I do not think that I am over-stating the picture, degrading as it is to our profession, for the last sentence is quoted from an author who knew London and its people as few other have, and his illustration may be relied on as truthful.

Of the practice of surgery in the Georgian Era I need say little. The revolution in that branch of our art is so recent that it will suffice to say that surgery, as practised then, was little in advance of what obtained in the days of the Roman Empire. Medicine was not in a much better position. The antiquated pharmacopœia was being thrust aside by more modern and rational drugs, and if some of the methods employed appear to us to be crude, if not barbarous, it may be said that they were the attempts, heroic at times, to discover how disease might be treated in a more rational manner. One of the first steps taken was the introduction of the practice of inoculation for small-pox, then one of the commonest and most fatal of diseases. Issues, setons, cupping and bleeding were all in full use: only the last two remain with us in an emaciated condition. Johnson, though a great dabbler in physic, was not an advocate of periodic bleeding as was then so much in fashion. His opinion was that by frequent bleedings you accustom yourself to an evacuation that Nature cannot perform for herself, and therefore cannot help you, should you, from forgetfulness or any other cause, omit it: so you may suddenly suffocate. For other periodic evacuations Nature can supply the omission. Dr. Taylor, with whom this conversation was held, said that he did not like to take an emetic for fear of breaking some small vessels. "Oh," said Johnson, blowing with high derision, "if you have so many things to break you had better break your neck and there's an end on't. You will break no small vessels."

An insight into the drugs that were used in those days is given me by a copy of the Pharmacopœia of St. Thomas's Hospital for 1772. This little book was used by my great-great-grandfather, one of George III's physicians, and in it are numerous pages of notes on diseases, and the remedies employed, in his handwriting. I cannot go at length into the measures he took to cure his patients, but the list of drugs shows how few are still in use, or perhaps I should say how greatly we have increased our list.

The number of drugs mentioned in Boswell's *Life* is very small: opium, squills, Peruvian bark, and tartar emetic constitute the total. Johnson took all of these, and in his last illness "Dr. James's compound medicines," whose ingredients appeared to him "to be inefficatious and trifling, and sometimes heterogeneous and destructive to each other." Dr. James, whom I shall mention presently, has still one powder known by his name, "Pulvis Antimonialis," which is so simple as hardly to come under Johnson's condemnation. It was the day of huge mixtures, of which the celebrated Mithradate, having opium as its most important ingredient, was the best known, it having seventy-three different drugs to compose its formula. The compound medicines of James that Johnson took exception to contained a very large number and totalled three hundred and thirty grains, of which four were of tartar emetic. It also contained thebaic tincture, a preparation of opium. Johnson said of it, "He that writes thus surely writes for show. . . . We will, if you please, leave this medicine alone. The squills have every suffrage, and in the squills we will rest for the present." Rhubarb was then a rare drug and highly prized, so that Sir Alexander Dick's present to Johnson was a very marked attention. It came originally from China by the caravan route to Russia once a year. The Russian Government had a monopoly till 1762, when the trade was thrown open, but the Russian Rhubarb Office was only abolished in 1863. In my father's work on the *History of Prices* I find that half an ounce cost 5s. in 1741, a very high price.

(*To be continued.*)

